# Severe Heterotopic Ossification After Revision Total Knee Arthroplasty: A Case Report and Review of the Literature

**DOI:** 10.5435/JAAOSGlobal-D-22-00053

**Published:** 2022-11-11

**Authors:** Dennis Vanden Berge, Kevin Bondar, Ramakanth Yakkanti, David Constantinescu, Jaime Alberto Carvajal Alba

**Affiliations:** From the Department of Orthopedic Surgery, University of Miami Hospital, Miami, FL (Dr. Vanden Berge, Dr. Bondar, Dr. Yakkanti, Dr. Constantinescu, and Dr. Carvajal Alba)

## Abstract

The incidence of primary and revision total knee arthroplasty (TKA) is increasing worldwide. Heterotopic ossification is a common and concerning complication of TKA. There are few described cases of severe heterotopic ossification after revision TKA and no known cases of heterotopic ossification causing functional ankylosis after revision TKA. We describe a case of extensive heterotopic ossification in a patient who underwent right TKA for extensive adhesions and stiffness. After early range of motion improvement postoperatively, the patient discontinued a physical therapy regimen. The patient presented 13 years after revision TKA with radiographically evidenced severe heterotopic ossification resulting in a functional ankylosis. The patient elected for nonsurgical management. This case demonstrates a delayed finding of severe heterotopic ossification. The case prompted an applied literature review of several topics: heterotopic ossification as a complication of revision arthroplasty, the contribution of autoimmune and inflammatory conditions to heterotopic ossification; the use of medication, radiation, and physical therapy as prophylaxis against heterotopic ossification; and the range of treatment strategies for severe heterotopic ossification at the knee joint. Consent by the patient involved in this case report was obtained.

Heterotopic ossification (HO) is defined as bone formation in atypical soft-tissue locations. Common etiologies include joint arthroplasty, muscle trauma, and spinal cord injury.^1^ HO commonly presents with localized tenderness, swelling, and decreased range of motion. HO may present with a slowly progressive mass. Typical radiographic findings include a soft-tissue mass, which progresses to peripherally radiating calcification, and eventual mature bone formation.^2^ Typically, HO is treated conservatively initially with surgical removal after maturation in symptomatic cases. In cases that occur after total knee arthroplasty (TKA), treatment has varied widely between conservative and surgical approaches.

Many surgical factors may predispose a patient to develop HO after TKA, including manipulation of soft tissues, femoral notching, soft-tissue stripping, retained bony fragments from bony resection, and knee effusion.^2^ Previous studies discuss patient functional status after HO development following primary TKA,^3,4^ but there are limited data discussing HO after revision TKA. This is the first documented case of HO after revision TKA causing a functional ankylosis.

The present study aims to characterize a severe case of HO, to comment on potential predisposing factors, to discuss treatment measures indicated for this patient, and to discuss the literature on HO after revision arthroplasty. The patient in this study consented to participate in this case report.

## Case History

The patient is a 67-year-old woman with a medical history of psoriasis, diabetes mellitus, and ductal carcinoma in situ of the left breast s/p left breast mastectomy with negative lymph node biopsies who presented to the outpatient orthopaedic joint reconstruction clinic with a chief complaint of bilateral knee pain. The patient originally underwent a right TKA at an outside hospital in 2007. The patient did not provide records from her original surgery and previous clinic appointments; therefore, the preoperative range of motion, surgical technique, and preoperative radiographs of the index procedure could not be reviewed.

Postoperatively, the patient developed adhesions and stiffness in the right knee requiring two manipulations under anesthesia. She developed significant stiffness and pain in the right knee with a flexion contracture causing inability to ambulate for roughly 1 year. In 2008, after a negative infectious workup, the patient underwent a right total knee revision arthroplasty at another outside hospital. Records of the preoperative and postoperative imaging and surgical reports were unavailable. Postoperatively, the patient completed aggressive physical therapy programs and improved range of motion to approximately 0° to 100°. She subsequently again developed flexion and extension contractures. Because of several insurance issues, the patient was unable to continue with recommended physical therapy. Her range of motion began decreasing after discontinuing physical therapy. At an orthopaedic clinic visit in 2011, the patient was noted to have mild diffuse tenderness to the right knee. The patient was able to ambulate with a cane. There were no signs of infection on examination. The right knee range of motion was 10° to 45°. An aggressive physical therapy regimen was recommended at this time.

In March 2021, 13 years after the revision TKA, the patient presented to the investigator's clinic for evaluation of left knee pain and inability to range the right knee. She denied pain to the right knee and was able to ambulate roughly two blocks without significant discomfort, but with a severe limp. Abnormal physical examination findings of the right lower extremity included the right knee locked at 5° of flexion and psoriatic plaques on the bilateral lower extremities. She had adequate patellar tracking and ligamentous balancing. Extensive heterotopic ossification of the right knee was noted on a radiograph. An infectious workup, conducted after both the primary and revision TKA, suggested that infection was not the etiology of HO formation. Her left (contralateral) knee showed abnormal varus alignment with pain most severe at the medial joint line.

**Figure 1:**
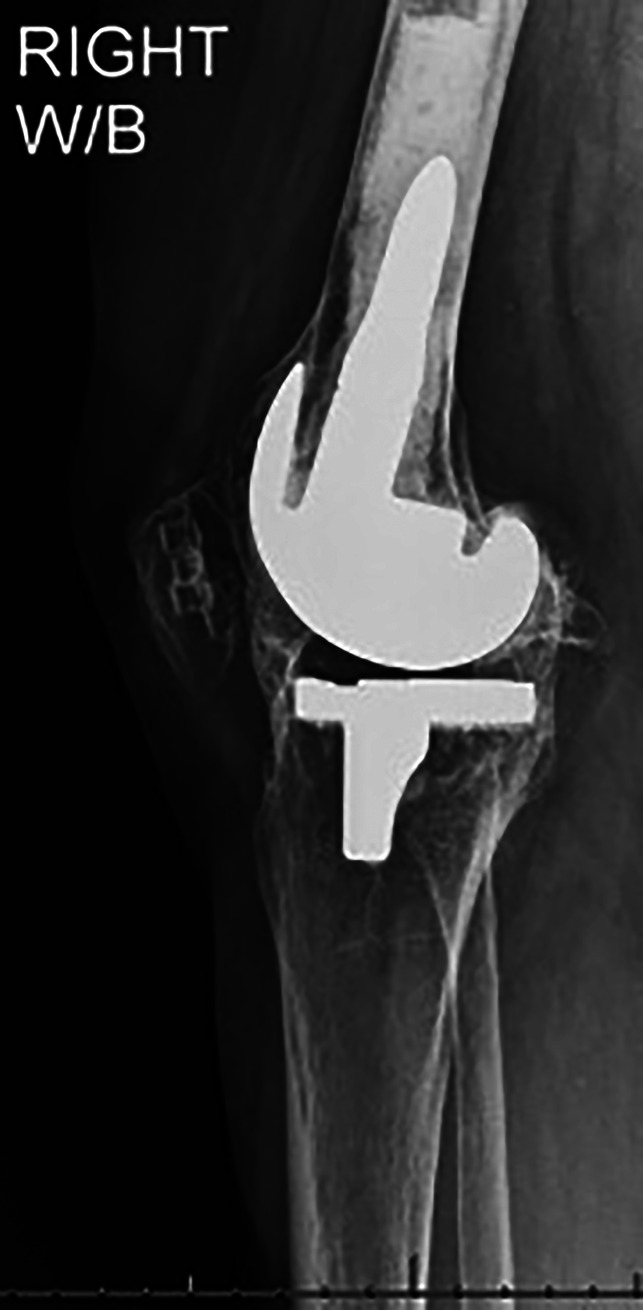
Lateral radiograph of the right knee.

**Figure 2:**
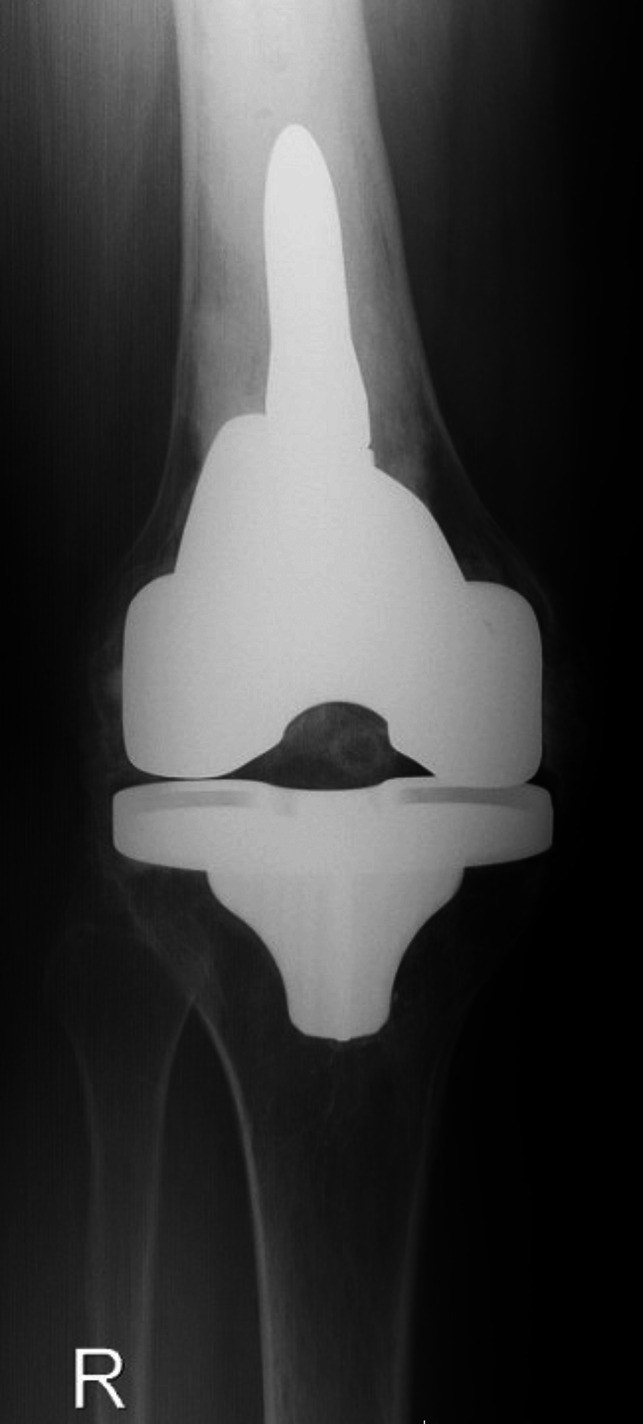
AP radiograph of the right knee.

**Figure 3:**
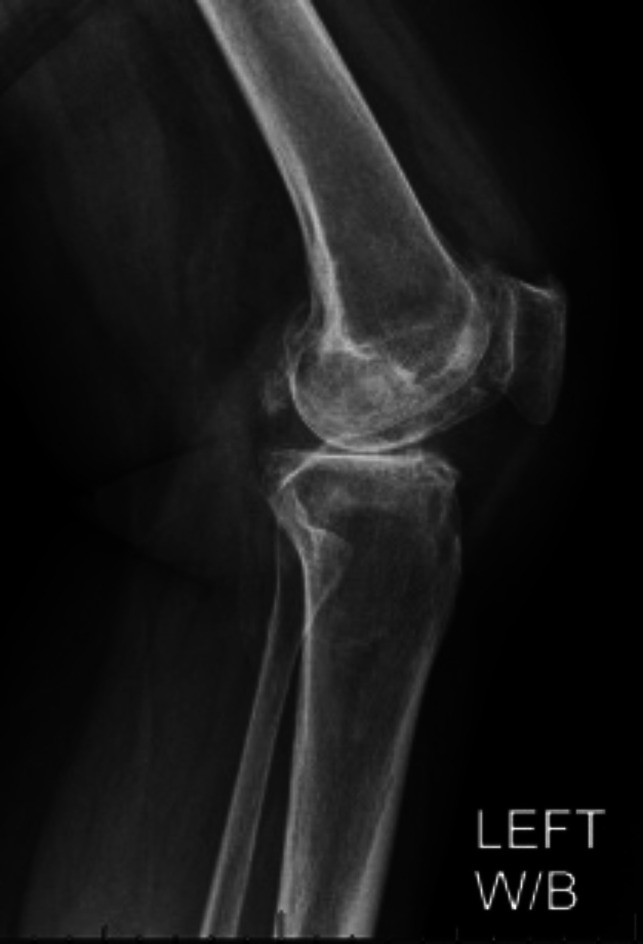
Lateral radiograph of the left knee.

Standing AP and lateral radiographs of the right knee revealed a right knee prosthesis in place with adequate component position and no signs of loosening or periprosthetic fractures. There was extensive heterotopic ossification involving the entire knee including the medial collateral ligament, lateral collateral ligament, anterior soft-tissues/capsule, and posterior capsule with bony bridging in medial and lateral compartments of the knee both anteriorly and posteriorly (Figures 1 and 2). Standing AP and lateral views of the left knee revealed tricompartmental osteoarthritis with joint line narrowing, worst in the medial compartment (Figure 3). There was no known ankylosis of other joints. Upper extremity imaging was not performed.

The patient visited rheumatology and spine clinics for lumbar back pain with left-sided radiculopathy. Sacroiliac (SI) joint and lumbar spine radiographs and MRI of the lumbar spine demonstrated spondylosis of L5-S1 and enthesopathic changes of the bilateral iliac crests. Dual-energy X-ray absorptiometry scan results were as follows: T scores of −0.9 (lumbar spine), −1.4 (left femoral neck), and −1 (left hip), indicative of osteopenia. C-reactive protein was 0.4, within normal limits. Rheumatoid factor was <10, within normal limits.

Long-term conservative treatment was recommended for treatment of her left knee osteoarthritis including physical therapy, anti-inflammatory medications, and injections. The patient received an unknown quantity of platelet-rich plasma injections for the left knee pain 10 to 15 years before presentation at the investigator's clinic. The patient has chronically taken the following: meloxicam 15 mg orally as needed, acetaminophen orally as needed, and 1% diclofenac gel as needed for both knees. The patient received one hyaluronic injection in November 2021 and noted symptomatic improvement at the follow-up visit in 2022.

Treatment options for the extensive heterotopic ossification of the right knee were discussed. The patient reported no pain and chose to pursue conservative treatment, with the understanding that her knee range of motion would likely not improve.

## Discussion

We present a case report of HO causing functional arthrodesis that arose after revision right TKA. Many reports have described HO after primary TKA, with incidence ranging as high as 39%.^5,6^ In primary TKA, HO was found to not have a major influence on functional outcomes and range of motion.^5^ There is a scarcity of literature describing significant HO after revision TKA. This is the first case report describing a complete functional arthrodesis of the knee joint after revision TKA.

Patients with severe HO after revision TKA have decreased range of motion postoperatively. Baroudi et al. described a case of a 67-year-old woman who developed significant HO after revision of the tibial implant after a periprosthetic tibial plateau fracture with associated implant loosening. At the 3-week postoperative visit, the patient developed maximum flexion of 70°, and extensive HO was appreciated on radiographic films. Five months later, the patient developed severe loss of motion with a maximal arc of motion of 20°. After maturation of the pathology, the patient underwent HO excision and revision to a segmental distal femoral implant and a rotating hinged knee modular tibial platform. At 1-year follow-up, the range of motion was from full extension to 100° of flexion.^3^ A prospective cohort study by Gkiatas et al.^7^ found that patients with more severe HO had decreased range of motion postoperatively, but there was no difference in postoperative range of motion patterns in patients with HO compared with those without HO. The present case is an example of severe HO with complete loss of range of motion.

Barrack et al. performed a case series evaluating the incidence of HO after revision TKA at two medical centers. There was an incidence of HO at 56% after revision surgery compared with 23% before the revision surgery. The presence of infection (76% compared with 47% HO in patients who did not have infection) was the sole significant risk factor identified. In patients developing HO, there were Knee Society functional outcomes scores (129 points versus 148 points). The average range of motion was 8° lower in patients who developed HO. This difference was not statistically significant.^8^ Case studies by Aljurayyan et al.^9^ and Manrique et al.^10^ report revision surgery after primary or revision total hip arthroplasty and the number of total hip arthroplasty procedures as independent risk factors for HO formation. Our patient developed severe functional limitations and progressively worsening range of motion. Infectious workup was negative and was likely not a contributing etiology.

The patient originally developed significant stiffness and adhesions after primary TKA, requiring multiple manipulations under anesthesia and subsequent revision. Gkiatas et al. evaluated the incidence of HO in revision TKA. Compared with patients undergoing revision for aseptic loosening, patients who underwent revision TKA for stiffness were found to have a higher incidence of HO before the procedure (30% versus 10.6%, *P* = 0.03). Patients with HO were also found to have decreased preoperative range of motion (52° compared with 63°), which improved at the final 1-year follow-up (81° vs 86°).^7^

Immunologic and inflammatory conditions may contribute to heterotopic ossification. The patient's comorbidities including diabetes and psoriasis may have contributed to heterotopic ossification. Patients with psoriasis can develop HO formation and develop flares in the region of the surgical scar, known as the Koebner phenomenon,^11^ similar to this patient's presentation. Despite a positive personal and family history of psoriasis, this patient did not meet the Classification for Psoriatic Arthritis criteria. Immunologic processes involving cytokines and chemokines, which are prominent in various rheumatologic conditions, contribute to the pathogenesis of heterotopic ossification and may be reduced with early treatment with biologic therapeutics.^12^ The patient had no known burns, other musculoskeletal trauma, or central nervous system injury, all of which can contribute to HO. Laboratory evaluation and sentinel lymph node biopsy demonstrated no recurrence or metastasis of ductal carcinoma of the breast. Carcinoma likely did not contribute to the right knee HO in this patient.

Stimulan beads may be an additional predisposing factor to HO after revision arthroplasty. Skeletal muscle acts as a nidus for bone formation with the use of calcium sulfate/hydroxyapatite material in Stimulan beads.^13^ Calcium sulfate can lead to locally increased calcium concentrations, increasing the risk of HO. HO formation has a multifactorial etiology.^14^ Kallala et al. performed a prospective study evaluating Stimulan beads in the use of revision hip and knee arthroplasty. Of note, 1.7% of revision cases developed HO. There was no statistically significant relationship between development of HO and the volume of beads (*P* > 0.05).^15^ In the cases of HO, HO was easily removed by secondary procedures. Whether the Stimulan beads and the revision procedure contributed to the HO and the potential influence of the etiology on the ease of resection of HO is unknown.

A viable treatment option for a patient with HO after revision arthroplasty may be procedural removal of the HO although functional outcomes after removal are a subject of future research. Physical therapy exercises, medications, manipulation under anesthesia, and surgical resection, and revision arthroplasty are key treatment options for HO after TKA.^4,16^ Prophylactic radiation^17^ and selective NSAIDs^18^ have shown benefit as prophylaxis against HO for patients undergoing TKA. There are serious risks of bony nonunion and carcinogenesis with these treatments. Novel prophylactic treatments include bone morphogenetic inhibitors, pulsed electromagnetic fields, and free radical scavengers and require further testing.^19^

There are limitations to the current case report. The patient reported first to the orthopaedic clinic 13 years after revision surgery with complete maturation of the HO. Preoperative function was described by the patient, but previous medical documentation was not provided during the clinic visit; therefore, the complete workup and findings before the clinic presentation cannot be fully described. Second, the patient presented to clinic to discuss the pain to her contralateral knee with tricompartmental osteoarthritis diagnosed on workup. The patient wished to continue conservative treatment for the HO; therefore, further workup was not pursued.

## Summary

The present case highlights a patient with a case of functional arthrodesis due to severe HO after revision arthroplasty. Revision arthroplasty surgery may be more likely to cause HO due to underlying increased inflammatory mediators and increased damage to bone and soft tissues. With decreased range of the knee post-TKA, HO should be suspected. Prophylactic radiation and medication may reduce HO formation. Conservative treatments or surgical removal of HO after revision arthroplasty are options. Treatment should be based on a combination of pain, range of motion, functional limitations, and HO severity and location. Consistent clinical follow-up and long-term physical therapy are recommended after revision arthroplasty.
